# Transcriptome analysis of antigenic variation in *Plasmodium falciparum - var *silencing is not dependent on antisense RNA

**DOI:** 10.1186/gb-2005-6-11-r93

**Published:** 2005-10-31

**Authors:** Stuart A Ralph, Emmanuel Bischoff, Denise Mattei, Odile Sismeiro, Marie-Agnès Dillies, Ghislaine Guigon, Jean-Yves Coppee, Peter H David, Artur Scherf

**Affiliations:** 1 Institut Pasteur, Unit of Biology of Host-Parasite Interactions, Centre National de la Recherche Scientifique, Unité de Recherche Associée 2581, 25 Rue du Docteur Roux, F-75724 Paris Cedex 15, France; 2 Institut Pasteur, Plate-Forme 2 - Puces à ADN, 28 Rue du Docteur Roux, F-75724 Paris Cedex 15, France; 3 Institut Pasteur, Unité d'Immunologie Moléculaire des Parasites, 28 Rue du Docteur Roux, F-75724 Paris Cedex 15, France; 4 The Walter and Eliza Hall Institute of Medical Research, 1G Royal Parade, Parkville, Melbourne 3050, Victoria, Australia; 5 Institut Pasteur, Plate-Forme 8 - CNR/Santé Publique, 28 Rue du Docteur Roux, F-75724 Paris Cedex 15, France

## Abstract

**Background:**

*Plasmodium falciparum*, the causative agent of the most severe form of malaria, undergoes antigenic variation through successive presentation of a family of antigens on the surface of parasitized erythrocytes. These antigens, known as *Plasmodium falciparum *erythrocyte membrane protein 1 (PfEMP1) proteins, are subject to a mutually exclusive expression system, and are encoded by the multigene *var *family. The mechanism whereby inactive *var *genes are silenced is poorly understood. To investigate transcriptional features of this mechanism, we conducted a microarray analysis of parasites that were selected to express different *var *genes by adhesion to chondroitin sulfate A (CSA) or CD36.

**Results:**

In addition to oligonucleotides for all predicted protein-coding genes, oligonucleotide probes specific to each known *var *gene of the FCR3 background were designed and added to the microarray, as well as tiled sense and antisense probes for a subset of *var *genes. In parasites selected for adhesion to CSA, one full-length *var *gene (*var2csa*) was strongly upregulated, as were sense RNA molecules emanating from the 3' end of a limited subset of other *var *genes. No global relationship between sense and antisense production of *var *genes was observed, but notably, some *var *genes had coincident high levels of both antisense and sense transcript.

**Conclusion:**

Mutually exclusive expression of PfEMP1 proteins results from transcriptional silencing of non-expressed *var *genes. The distribution of steady-state sense and antisense RNA at *var *loci are not consistent with a silencing mechanism based on antisense silencing of inactive *var *genes. Silencing of *var *loci is also associated with altered regulation of genes distal to *var *loci.

## Abstract 2

A microarray analysis of *Plasmodium falciparum *selected to express different *var *genes suggests that antisense transcripts are not responsible for the transcriptional silencing of non-expressed *var *genes.

## Background

*Plasmodium falciparum *is a parasite belonging to the phylum apicomplexa, a group characterized by intracellular parasitism. A striking feature of apicomplexans' intracellular lifestyle is their ability to modify host cells though export of macromolecules. *P. falciparum *parasitizes erythrocytes, which it proceeds to alter via the secretion of a large number of proteins. Much of this protein content is represented by the *Plasmodium falciparum *erythrocyte membrane protein 1 (PfEMP1) molecules, ligands that span the erythrocyte membrane and mediate cytoadhesion to human receptors exposed to circulating parasites. PfEMP1 proteins are encoded by *var *genes, and field isolates possess approximately 60-70 distinct *var *genes. Each *var *gene consists of a large variable 5' exon (around 4-9 kb in length), and a smaller, more conserved 3' exon (around 1 kb in length) that encodes the intracellular portion of the PfEMP1 protein. Individual parasites do not express all PfEMP1 isoforms simultaneously, but rather change from one *var *to another successively. The adaptive pressure that selects such behavior is controversial, but plausible hypotheses include avoidance of host antibody response, and changes in cytoadherence ligand in response to tissue environment.

Switching of transcription from one *var *gene to another does not require genetic rearrangements [1,2] (unlike antigenic variation in *Trypanosoma brucei*), but is instead associated with epigenetic changes [3-5]. Parasites can change from expressing one PfEMP1 molecule to another both *in vivo *and *in vitro*. The rate at which parasites switch away from their parental phenotype is difficult to measure, and different methods have resulted in estimates varying from less than 1% per generation *in vitro *[6], to more than 16% per generation *in vivo *[7].

The switching of active *var *genes *in vitro *means that cloned parasites expressing individual *var *genes will eventually drift in the absence of immune pressure to heterogeneous populations. This makes it difficult to assess how many *var *genes are being expressed in individual parasites. However, parasites selected for binding to different host receptors express distinct *var *genes and such populations have previously been described to transcribe single dominant *var *genes [2]. Nevertheless many contentious questions remain about how *var *genes are transcriptionally regulated. Some studies have suggested that mutually exclusive expression is developmentally controlled, with a number of *var *genes being transcribed in ring-stage parasites, but only a single *var *transcribed in the later trophozoite stage [2,8]. Other studies suggest that transcription is initiated at a number of *var *loci, but that only a single *var *gene produces complete transcripts [9]. Another puzzling phenomenon is the so-called sterile transcripts that are apparently produced from the 3' exon of many *var *genes [10].

Analysis of the *var *introns shows that they contain a promoter that is responsible for the sterile transcripts. The same cryptic promoter was also shown to be bi-directionally functional in reporter assays [11], raising the intriguing prospect that antisense transcripts may play a role in *var *regulation. Antisense transcription has been suggested as a general control mechanism for *Plasmodium *transcription [12-14], with a global transcription profile indicating an inverse correlation between abundance of sense and the ratio of sense-to-antisense for many loci. Additionally, artificially introduced antisense molecules have been used to specifically downregulate some genes in *P. falciparum *[15-17]. Widespread antisense transcripts are also believed to be involved in the modulation of gene expression in humans [18], rice [19], and *Arabidopsis *[20]. Although antisense is commonly seen as a means of downregulating expression of the protein-coding strand, several global transcriptional studies indicate some sense and antisense RNAs are co-regulated, with transcription of both strands up- or downregulated in certain conditions or tissues [21,22].

To address these important outstanding questions concerning regulation of *var *genes we constructed a customized oligonucleotide array containing sense and antisense probes to all known *var *genes of the *P. falciparum *FCR3 strain, in addition to probes to all other predicted protein coding genes of the sequenced 3D7 strain [23]. Individual parasites have approximately 60 *var *genes, and of these, 36 have been identified so far in FCR3. For a subset of eight *var *genes, we made tiled probes against both strands, spanning from the 5'UTR to the 3'UTR. Parasites were panned on either CD36 or chondroitin sulfate A (CSA) to select for parasites expressing distinct *var *genes, then compared at three points through the asexual intraerythrocytic life cycle. We hypothesized that upregulation of a *var *gene would be accompanied by decreased abundance of complementary antisense, while downregulated *var *genes would be associated with an increase in corresponding antisense RNA. Instead, we found that no consistent positive or negative correlation existed between abundance of sense and antisense transcripts. Notably, the very strong upregulation of *var2csa *gene (Genbank: AY372123) in CSA-selected parasites was accompanied with substantially increased abundance of antisense RNA throughout the same gene. These data indicate that antisense RNAs do not control antigenic variation in *Plasmodium*. We failed to find any evidence for *var *transcripts that included only the 5' end, and we also show that 3' sterile transcripts are limited to a subset of *var *genes.

Parasite adhesion phenotypes also correlate with some specific patterns of physiopathology so other non-*var *genes upregulated in association with specific binding types are of interest. We detected several genes that are differentially transcribed between CSA and CD36 parasites, including mature parasite-infected erythrocyte surface antigen (MESA - known to bind to the erythrocyte membrane cytoskeleton) and other proteins predicted to be exported to the infected erythrocyte.

## Results and discussion

### Transcriptional changes in *var *genes

**Figure 1:**
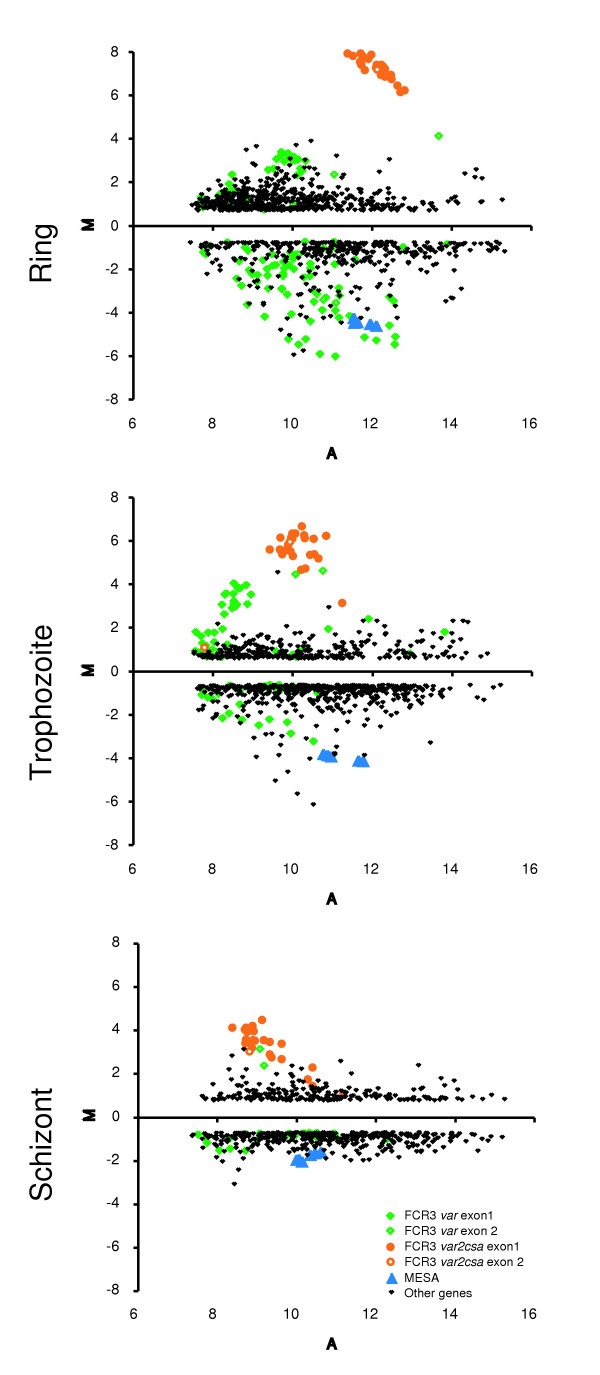
A dominant *var *gene is upregulated in CSA binding parasites. Plots of log2 ratio of expression (M) against average log intensity (A) for ring, trophozoite and schizont stages for CSA versus CD36 panned parasites. Only statistically differential data giving a Bonferroni corrected *p* value (alpha = 0.05) have been displayed. This graph excludes probes corresponding to antisense transcripts and oligos to 3D7 *var *genes (whose orthologs in FCR3 diverge in sequence). Biological replicates were pooled. The plots reveal a single dominant *var *transcript (*var2csa*-marked in orange) that is much more abundant in CSA than in CD36-panned parasites at all life stages. Green dots represent all other oligos corresponding to FCR3 *var* genes. Several *var *genes are over-represented in CD36 as compared with CSA-panned parasites. Both log2 ratios of expression and apparent average intensities for *var *genes decrease through the life cycle.

**Figure 2:**
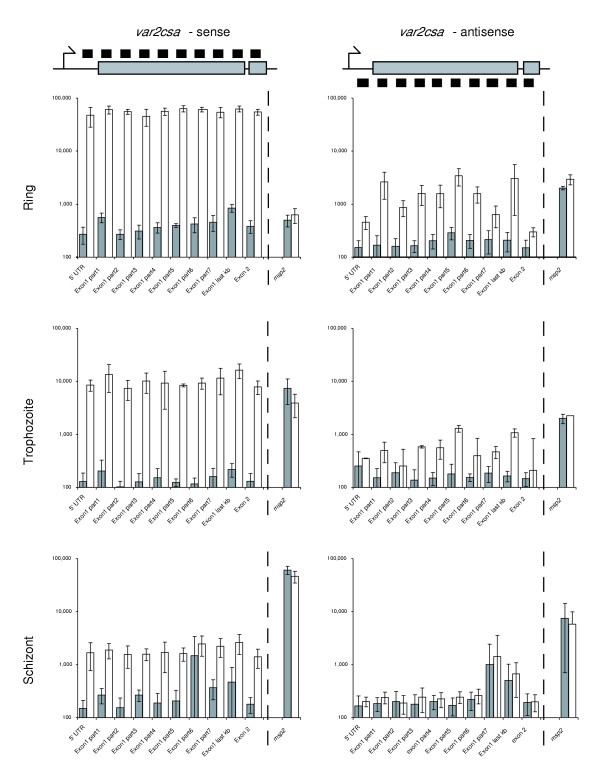
Consistent sense transcript and interspersed antisense transcript in *var2csa *gene. Histograms showing apparent absolute abundance of both sense and antisense transcript at the *var2csa* locus in CD36 (grey) and CSA (white) panned parasites. Different columns show the apparent absolute abundance for oligonucleotides at individual positions along the whole *var2csa *gene. Left panels show probes corresponding to sense transcript, right panels show probes corresponding to antisense transcripts. Separate histograms show data for ring, trophozoite and schizont stages. Standard deviation is shown. No truncated 5' transcript of the *var2csa *gene is apparent in CD36 panned parasites, suggesting regulation is not controlled by premature termination of transcription. In ring stages, where *var2csa *transcript is most abundant in CSA parasites, apparent absolute abundance is also increased for antisense transcripts throughout the gene. Unlike sense transcription, apparent absolute abundance for all antisense transcripts varies greatly between adjacent probes, perhaps indicative of multiple short antisense transcripts initiating throughout the locus. Abundance of sense and antisense transcript in both populations is also shown for a non-*var *locus, *msp2*, for which high antisense transcription has previously been measured [34]. Both steady-state sense and antisense levels for the *var2csa *locus are comparable with those found at the *msp2 *locus.

Arrays containing specific *var *gene probes for the FCR3 strain allowed us to assay steady-state RNA changes between CSA-panned and CD36-panned parasites. Total RNA was harvested from three time points through the parasite life cycle, at 12 hours, 24 hours and 36 hours post invasion. Parasites from these time points are referred to as ring, trophozoites and schizont stage parasites, respectively. Previous analyses have shown that the peak of *var *transcription is in ring stages [24,25] and this was confirmed by our analysis, with highest total *var *transcripts present in ring stages (Figure 1) for both FCR3-CSA and FCR3-CD36. A comparison of the two populations revealed that several *var *genes are expressed in the FCR3-CD36 population, while only one dominant *var*, known as *var2csa *(or PFL0030c) is apparent in the FCR3-CSA population. Multiple probes from this gene detected transcripts at an abundance 150 to 200-fold higher in FCR3-CSA than in FCR3-CD36 parasites (Figures 1 and 2). This could reflect the almost total absence of *var2csa *transcripts in FCR3-CD36 parasites. Peak transcript abundance for this gene was in ring stages, with the fold-difference between populations falling markedly in trophozoite (60 to 80-fold) (Figure 2) and schizont parasites (6 to 10-fold) (Figure 2). Only hybridization ratios and not levels of hybridization are appropriate to consider when interpreting results obtained with this type of glass spotted microarrays. However, the absolute values obtained for each RNA population (we will refer to these as 'apparent absolute transcript levels' or AATLs), also strongly suggest that peak transcript abundance for this gene was in ring stages. Considering all *var *and non-*var *genes, *var2csa *was the most highly upregulated gene found in FCR-CSA relative to FCR3-CD36 and had one of the highest AATLs detected in these parasites (Figure 1). These data are consistent with previous reports that find a correlation between CSA binding and expression of *var2csa *in different strains [26-28]. Northern analysis of FCR3-CSA and FCR3-CD36 parasites prepared in our laboratory also shows a very high expression of *var2csa *in CSA binding parasites and none in CD36-binding parasites [29]. Cross-reactive probes directed against *var *exon 2, which detect most (but not all) *var *genes detect no other *var *transcripts in CSA-binding parasites [29]. Additionally, FCR3 parasites with the *var2csa *gene disrupted can no longer bind to CSA. Although our array covers all currently known *var *genes for the FCR3 strain, not every *var *gene has been sequenced. We therefore cannot exclude that another unknown *var *gene is involved in CSA binding, although evidence from transcription, proteomic, serological and biochemical studies now indicates that upregulation of *var2csa *is central to CSA binding [26,27].

In addition to the major *var2csa *transcript, the microarray analysis detected a less pronounced upregulation of a second full-length *var *transcript in the CSA-binding population - the *A4-tres *gene. The probes corresponding to this open reading frame (ORF) indicated a 5 to 9-fold upregulation of this gene in FCR3-CSA parasites compared with FCR3-CD36, but the AATL for this gene is still relatively low (Additional data file 1), and *varA4-tres *transcript is not detected in CSA-panned parasites by Northern blot using cross-reactive *var *probes [29]. The A4tres protein is unable to mediate CSA binding in *var2csa *knockout parasites, so it is unclear whether A4tres has a role in CSA binding.

Unlike CSA binding, multiple *var *genes are known to participate in CD36 interactions [30]. It is therefore unsurprising that several *var *genes are upregulated in the FCR3-CD36 population (Figure 1, Additional data files 1 and 2). No *var *gene in this population exhibits the same fold change or the same AATL as the *var2csa *gene in FCR3-CSA. This suggests that the FCR3-CD36 population is not homogenous, but rather a heterogeneous mix of parasites each expressing one of a select subset of *var *genes. The molecular basis for CD36 binding is relatively well understood, and the domains responsible for the interaction have been identified in several strains [31-33]. The upregulated *var *genes in FCR3-CD36 include domains that have been previously demonstrated to encode CD36-binding PfEMP1 proteins (for example, *varFCR3S1.2*), as well as several poorly characterized *var *genes (for example, *var_clone_70, var_cDNA11*).

The current paucity of panning systems for selecting monomorphic populations prevents us from determining if the behavior of the *var2csa*-expressing parasites is representative of all *var *types. Both the characterization of additional receptor-ligand interactions and the development of selectable markers in or adjacent to *var *genes should generate valuable tools to address this in the future.

### Antisense RNAs

Global and specific transcriptional profiles of *P. falciparum *indicate extensive transcription from the antisense strand of many genes [12]. Nuclear run-on assays show that antisense production is highly alpha-amanitin sensitive, implying a dependence on RNA polymerase II activity [14]. As in some other organisms, the distribution of *Plasmodium *antisense transcripts suggests a role in regulation of sense strands, with abundance of sense and antisense frequently inversely related for given loci [13]. The availability of genes specifically up- or downregulated at the same life stage, and in genetically identical parasites, creates an ideal system to test the importance of antisense RNAs for *Plasmodium *gene expression. To investigate this mechanism, we designed specific oligonucleotides probes for antisense RNAs derived from all known *var *genes of the FCR3 strain. For eight of these genes we also printed multiple oligonucleotide probes tiling the sense and antisense strands of eight different *var *genes (see Additional data file 1). These include *var *genes strongly upregulated (*var2csa*), weakly upregulated (*varA4tres*), downregulated (*varFCR3S1.2*) or with no change (*varITOR29, varITO4*A4) in FCR3-CSA relative to FCR3-CD36.

**Figure 3:**
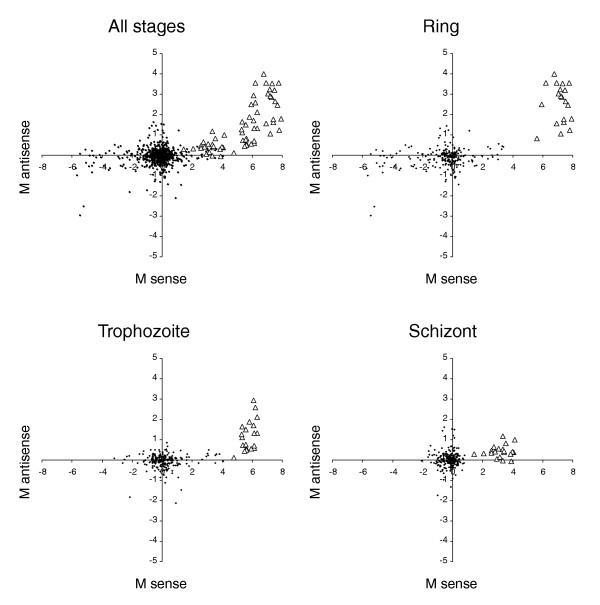
No inverse correlation between sense and antisense ratio changes. Scatter plots of log2 ratio of expression (M) (CSA-panned parasites over CD36-panned) for antisense oligonucleotides against sense oligonucleotides for *var *genes. Data are shown for ring, trophozoites and schizont stages from biological replicate 1. Oligonucleotides corresponding to *var2csa *are represented by open triangles and the other *var *genes from the FCR3 strain are displayed as black dots. Oligonucleotides with the highest log2 ratio of expression in CSA- compared with CD36-panned parasites often correspond to those with the highest corresponding ratios for antisense abundance (upper right datapoints). Similarly, several sense transcripts apparently highly upregulated in CD36 correspond to upregulated antisense oligos at the same loci (lower left datapoints). These data are not consistent with a direct transcriptional silencing role for antisense transcription.

Our data reveal a pattern for *var *antisense transcripts that is not consistent with direct antisense transcriptional inhibition (Figure 3). For *var *loci with high upregulation of sense transcript, the corresponding antisense was sometimes downregulated and sometimes upregulated. Similarly, downregulation of some sense transcripts was seen in conjunction with downregulation of complementary antisense but for other *var *genes was accompanied with upregulation of antisense (Figure 3). It is noteworthy that for the most highly upregulated sense transcripts (for example, the *var2csa *gene in CSA panned parasites), strong upregulation of antisense was also seen (Figure 2). The abundance of these antisense molecules is comparable with that produced from other genes known to have highly abundant antisense (for example, MSP2 [34]) (Figure 2). For *var *loci, these antisense RNA molecules were distributed throughout the gene, although their apparent absolute abundance was much more variable than that of the corresponding sense strand. For example, sense probes throughout the *var2csa *gene detected consistently strong upregulation throughout the ORF, while antisense RNAs were highly upregulated at some positions in the same gene and not at all in other positions (Figure 2). The large changes in both apparent absolute abundance, and in fold change for neighboring probes against antisense, suggests that antisense RNAs may not be large molecules spanning the entire gene, but rather multiple short transcripts initiating and terminating several times within several kb. Although promoter elements in *var *introns have been described that appear to drive reverse strand transcription (at least on plasmids) [11], the scattered production of antisense RNA that we observe points to weak promoter-like activity dispersed throughout the *var *genes. Our failure to detect antisense for the *var *loci that are silenced does not conclusively prove that they cannot play a role in *var *silencing, but the presence of abundant antisense molecules that coincide with highly transcribed (and translated) mRNA molecules strongly argues against their having a direct role in gene silencing.

**Figure 4:**
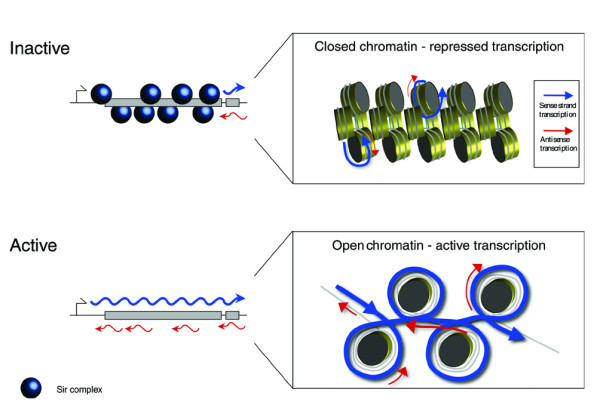
A hypothetical model for antisense transcription from *var *loci. Sense and antisense RNA at several *var *loci appear to be coordinately regulated. This may result from the altered chromatin state of the encoding genomic DNA, which is differentially modified between silent and active *var *loci [3]. Silencing factors such as the SIR complex (indicated by blue spheres) bind to inactive *var *genes, maintaining the chromatin in a condensed state. In the absence of SIR, the active *var *assumes a relaxed chromatin conformation that makes the surrounding locus competent for transcription. While a stable transcription complex with appropriate assembly of elongation factors generates abundant sense mRNA of full length, transcription from the opposite strand initiates and quickly terminates to produce fragments of antisense. Simultaneous transcription of the same bases from opposite directions is unviable, but in a population, both transcription events may occur at the same time. A chromatin barrier located in the intron [11] may maintain the first exon in a silencing conformation while allowing relaxation of the second exon, leading to partial 3' transcripts from a subset of otherwise silenced *var *genes.

Both the interspersed distribution of antisense RNA molecules and their coincident high abundance with a strongly upregulated protein-coding gene are evocative of a non-specific induction that can correspond with activation of a *var *gene. Our current understanding of *var *gene activation is that *var *genes are activated through disassociation from silencing molecules, subsequent local histone modification and decondensation of the local chromatin environment [3-5]. Indeed this has been shown for the *var2csa *gene itself. Such modifications make the DNA more accessible to initiation factors and to RNA polymerase. This increased accessibility is consistent with the concept of relaxed non-specific transcription from both strands in the surrounding environment. We hypothesize that the production of antisense RNA, at least in the case of *var *genes, is not a mechanism for silencing the protein coding strand, but is rather a consequence of an open chromatin configuration and greater concentration of transcription factors required for expression of the active *var *gene (Figure 4). A similar explanation has been advanced for some human loci, where sense and antisense RNAs are co-ordinately regulated [22]. Long transcripts simultaneously produced from both strands are physically implausible, as one polymerase complex would displace the other. This is consistent with our finding that antisense fragments appear to be small, or alternatively, that sense and antisense are produced simultaneously but in different cells.

### Full length or incomplete transcripts?

Various studies of *var *transcription have been able to detect transcripts corresponding to multiple *var *genes from parasite populations [2,8] or from single cells [35]. Most of these studies have used degenerate primers targeted to the conserved DBL region found at the 5' of most *var *genes. These results have led to the widespread understanding that transcription initiates at many *var *genes, but full-length *var *genes are produced from only one or very few loci [9]. Unfortunately the size of these molecules has never been thoroughly investigated and we find no data in the literature to suggest that these RNA species are in fact prematurely truncated. Indeed where RT-PCR has been used to assay transcription of the 3' end of *var *genes (across the splice site) multiple transcripts are still detected, even in adhesion-restricted lines [36]. Certainly, sensitive RT-PCR amplifications do produce evidence of multiple *var *transcripts, but these multiple transcripts are undetectable by Northern analysis. Our data do not support the existence of truncated 5' transcripts resulting from multiple *var *loci, although it is possible that some transcript exists below the limits of detection. Additionally, our experiments are unable to address whether some transcripts from multiple loci might be produced but very quickly degraded. This is still a possible additional means of *var *regulation, although the only published nuclear run-on experiments (which can still only partially address this issue) found no evidence of 'leaky' transcription from multiple *var *loci [2].

Although there are no quantitative data available regarding the existence of truncated transcripts originating at the 5' end of *var *genes, Northern blots using a probe from the 3' exon do consistently detect abundant RNA, often referred to as 'sterile transcript'. These probes cross react with the large *pf60 *family of genes and pseudogenes, which are transcribed in late-stage parasites and are approximately 3 kb in length. Other transcripts of around the same size appear to emanate from *var *introns themselves [10], though it is unknown at which stage these intron-derived fragments are produced. These intron-derived fragments, and perhaps *pf60 *transcripts too, may be involved in *var *silencing. Assays conducted with luciferase reporter driven by a *var *promoter indicated that the presence of a flanking *var *intron is required for proper silencing [11]. Mutations perturbing the promoter activity within this intronic sequence also disrupt silencing, indicating the sterile transcripts may themselves play a role in *var *silencing. We investigated the distribution of these *var *intron-derived transcripts using *var *genes for which we had probes for exon 1 and exon 2 transcripts. Our data show that transcripts do originate from the *var *introns, but only for a subset of *var *genes. For several *var *genes in the FCR3-CSA parasites, probes throughout exon 1 indicate the gene is silenced, but exon 2 is strongly upregulated. For example, exon 1 of *varFCR3S1.2 *is downregulated 5 to 25-fold in FCR3-CSA parasites, but exon 2 probes show a 10 to 25-fold upregulation. For other silenced *var *genes (for example, *var2csa *in FCR3-CD36 parasites or *varFCR3 T11-1 *in FCR3-CSA parasites) no sterile transcript is apparent in the same parasites, nor is it upregulated at any of the life-stages sampled. For some loci, intron-derived transcript was most abundant in ring transcripts, while at other loci exon 2 transcript was more abundant in later-stage parasites (Additional data file 1). The confusing overlap and cross hybridization of the *var *exon 2 transcript with *pf60 *transcript makes it difficult to clarify the relative abundance of either RNA species by Northern blot.

The absence of sterile transcripts corresponding to some silenced genes indicates that continuous presence of sterile transcript is not an absolute requirement for *var *silencing. Calderwood and colleagues have speculated that the promoter for sterile transcripts may participate in silencing by acting as a buffer for chromatin spreading [11]. Alternatively, sterile transcripts may flag complementary genomic regions as targets for chromatin condensation. If either of these possibilities is true, the promoter activity might be required to initiate the silencing chromatin state, but not to maintain it. Our discovery that transcripts are produced from the introns of some silenced *var *genes but not others requires a rethinking of the involvement of sterile transcript in silencing.

### The *var1csa *gene

One *var *gene that has been implicated in CSA adhesion through serological and binding assays is the *var1csa *gene [37-39]. Consistent with recent reports [35,40], we find that this gene does not appear to be upregulated at a transcriptional level in CSA-binding parasites. A previous study indicated that this gene is transcribed throughout the erythrocytic life cycle, apparently irrespective of adherence phenotypes [40]. This pattern is confirmed by our data, which show apparently continuous low-level expression of the *var1csa *gene in both CSA- and CD36-panned populations (Additional data file 1). Our data do not exclude a role for the Var1CSA protein in CSA binding, but they do suggest that the transcription status of *var1csa *is not in itself indicative of CSA binding.

### Steady-state RNA changes in non-*var *genes

**Figure 5:**
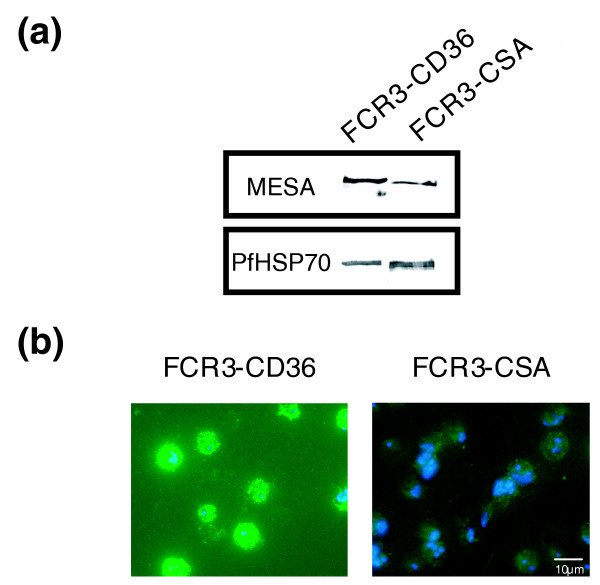
MESA overexpression in CD36 parasites. **(a) **Western blot of non-synchronized parasites from FCR3-CD36 and FCR3-CSA parasites. PfHsp70 protein is included as a loading control. A monoclonal antibody (Pf12.8B7.4) against MESA [60] detects approximately 2-4 times more protein in CD36 compared with CSA panned parasites. **(b) **Immunofluorescence for MESA protein in FCR3-CD36 and FCR3-CSA parasites. The 488-labeled secondary shows that MESA is considerably more abundant in CD36-compared with CSA-panned parasites. The intracellular distribution of MESA is the same in both parasite populations - with most labeling localizing to the periphery of infected erythrocytes.

Several non-*var *genes encoding parasite proteins predicted to be exported to the infected erythrocyte [41] are differentially abundant in our analysis (Additional data file 1). The most dramatic difference is seen for the *pfe0040c *gene, encoding the mature parasite-infected erythrocyte surface antigen (MESA - also known as PfEMP2). Three independent probes consistently registered 16-24 times greater abundance of this transcript in ring and trophozoite stages of the FCR3-CD36 parasites compared with FCR3-CSA (Figure 1). It is worth noting that MESA seems to be negatively co-regulated with *var2csa *(mean of Pearson R = -0.87 for a *var2csa *random sample of 6 of 30 values for each time point with the 6 *mesa *values available with 10,000 iterations). This was confirmed by Western blot (Figure 5a) and immunofluorescence (Figure 5b) with a monoclonal antibody specific for the MESA protein. Substantially more MESA is present in FCR3-CD36 than in FCR3-CSA parasites. The localization of MESA is unchanged between the two parasite types, with immunofluorescence showing a distribution at the erythrocyte periphery. In both populations, over 95% of mature parasites are positive for MESA by indirect immunofluorescence assay, so differences in transcript abundance are not due to a gene deletion in FCR3-CSA (as can sometimes happen with subtelomerically-located MESA). MESA is known to bind to the erythrocyte membrane skeletal protein 4.1 [42], and is thought to alter host cell membrane stability. However, erythrocytes infected by mutant parasites lacking MESA are able to adhere normally to CD36-presenting cells [43,44], indicating MESA is not required for cytoadhesion, at least *in vitro*. This does not exclude a role *in vivo *and the observation of major differences in levels of MESA expression between parasites expressing PfEMP1 with different adhesive properties is intriguing.

Transcripts representing several hypothetical proteins are differentially abundant in FCR3-CSA and FCR3-CD36, and their localization and function deserve further attention. Several possess targeting motifs predicted to direct their export out of the parasite and into the red blood cell (RBC) [41] (notable examples include PFC1080c, PFA0615w and PFD0080c) (Additional data file 1). Other annotated genes that are differentially regulated include the exported RBC protein GARP, and MAEBL, a predicted invasion ligand. The differential expression of genes not involved in cytoadhesion suggests that receptor use may actually trigger other changes that might be more involved in adaptations to tissue environment or local pH. Our data do not reveal any obvious candidates for signaling molecules involved in detection of or reaction to the parasites' external environment.

## Conclusion

The past three years have seen an increasing number of global transcriptional analyses of *P. falciparum*. Experiments have compared transcriptional changes between the vertebrate life stages [23,45], between genetically distinct strains [46,47], and in response to drug treatment [48] or glucose deprivation [49]. Despite high-quality, reproducible data demonstrating that a very high proportion of genes are rigidly and specifically regulated, recent reviews highlight our scant understanding of transcriptional control in *Plasmodium *[50,51]. Very few transcription factors have been identified, and genetic regulatory elements are not well described. This deficit has suggested to some that gene regulation in *Plasmodium *is post-transcriptionally controlled, perhaps by antisense-mediated repression [13].

Our analysis of parasite cytoadhesion shows that differences in receptor use are associated with limited specific transcriptional differences for both *var *and non-*var *genes. We find no changes in known transcription factors that associate with the observed differences. This is consistent with previous studies, which suggest that *var *transcription is regulated by histone modification and chromatin condensation. Silencing of *var *genes was not associated with increased antisense production at silenced loci, but rather, antisense abundance was in some cases coincident with high sense strand transcription. This indicates that *var *regulation is not mediated by antisense inhibition. Instead, antisense transcription may be a product of relaxation in the local chromatin structure (as reported in [3] and [5]), accompanied by loci moving to pro-transcription nuclear zones that may allow promiscuous conditions for transcription [3]. High-resolution microarrays offer very promising avenues for the investigation of such interactions between chromatin-mediated events and transcriptional regulation. Future studies will reveal DNA regions that are controlled by chromatin remodeling factors by superimposing array transcriptional information over data from 'ChIP-on-chip' analyses that use microarrays of immunoprecipitated chromatin to map specific chromatin features to the genome.

## Materials and methods

### Parasite culture

FCR3 parasites were cultured using modifications to the method described by Trager and Jensen [52]. Parasites were grown in a gas environment of 5% CO_2_, 1% O_2 _and 94% N_2_. Media was supplemented with 5% v/v human serum and 5% v/v Albumax II (Invitrogen SARL Cergy Pontoise, France).

### Panning of infected erythrocytes

*P. falciparum *strain FCR3 was panned on endothelial cells expressing either CSA (SBEC-17 line) or CD36 (SBEC-CS2 line) as described previously [2]. The resulting populations are hereafter referred to as FCR-CSA and FCR-CD36, respectively. Panning was repeated twice more, and parasites were tested for their ability to bind purified CSA (Sigma) or soluble recombinant CD36 (Affymax Research Institute) immobilized with monoclonal antibody 179 (Affymax Research Institute). After panning, parasites were expanded for 4-6 generations to generate sufficient quantities for analysis. Mature stages were eliminated using 0.3 M alanine in 10 mM HEPES [53]. Parasites were allowed to reinvade and were synchronized with 0.3 M alanine twice with an interval of eight hours to obtain tightly synchronous parasites. Parasites were allowed to reinvade once again, and were harvested at 12 hours, 24 hours and 36 hours post invasion. FCR3-CD36 parasites appeared to have a slight but consistently shorter life cycle than the FCR3-CSA parasites. For this reason, the schizont stage comparison was slightly asynchronous (2-4 h) with the CD36 parasites slightly more mature than the CSA. Subsets of parasites were assayed for their adhesion to CD36 and CSA immediately before and after each harvesting to confirm specificity of binding. Non-specific binding was at the level of the bovine serum albumin negative control for all populations.

### Total RNA preparation

Infected erythrocytes were washed in PBS, permeabilized with 0.05% saponin in PBS, washed three times in PBS, and lysed in 10 pellet volumes of Trizol (Gibco) before freezing at -80°C. Total RNA was prepared from thawed samples as per the manufacturer's instructions. RNA quality was assessed with an Agilent 2100 Bioanalyser (Additional data file 4).

### Oligonucleotides

The Malaria Oligo Set (Qiagen-Operon), designed by DeRisi [54], containing 7,393 optimized 70-mers corresponding to 4,644 annotated genes and to putative ORFs, was completed with 1,477 new oligos we designed using ArrayOligoSelector [54,55]. These new oligonucleotides corresponded to annotated genes in PlasmoDB that lacked oligos in the set, and also, sense and antisense probes to all known *var *genes of the *P. falciparum *FCR3 strain; for a subset of *var *genes, tiled probes were designed against both strands, spanning from the 5'UTR to the 3'UTR.

### Microarray spotting, cDNA target labeling hybridization and scanning

Oligonucleotides were resuspended in 3X SSC at 40 μM and printed onto UltraGAPS glass slides (Corning) using a Chipwriter Pro Virtek arrayer (Biorad). After printing, arrays were treated as per the instructions of the slide manufacturer (Corning).

RNA samples (5 μg) were indirectly labeled using Atlas PowerScript Fluorescent Labeling kit (Clontech) with a mixture of random hexamer (pdN6), according to the conditions recommended by the manufacturer, with the following modifications: after reverse-transcription, RNA was digested with RNAse H for 45 minutes at 37°C. cDNAs were coupled with cyanines using Cy3 Mono-Reactive Dye or Cy5 Mono-Reactive Dye (Amersham Bioscience). Fluorescent cDNA was then purified with QIAquick PCR Purification Kit (Qiagen). Target quality and concentration were determined by spectroscopy at 260 nm, 280 nm and 550 nm (Cy3) or 650 nm (Cy5). Cy3 and Cy5 target quantities were normalized at 250 pmol, mixed and thereafter concentrated by Microcon YM-30 (Millipore). Sample volumes were adjusted to 50 μl in 5X SSC, 0.1 mg/ml fragmented Salmon sperm DNA (Sigma), 30% formamide and 0.1% SDS.

Microarrays were pre-hybridized in 5X SSC, 1 mg/ml BSA and 0.1% SDS for 1 hour at 42°C, and then washed by immersion in dH2O for 1 minute, followed by isopropanol and dried by centrifugation for 2 minutes at 1,500 rpm. Fluorescent targets were denatured 3 minutes at 95°C, incubated at RT for 5 minutes prior to hybridization and briefly spun, then loaded onto the array under a LifterSlip (Erie Scientific) and incubated in a humid chamber (Telechem) for 16-18 hours at 42°C. After hybridization, slides were washed twice in 2X SSC and 0.1% SDS at 42°C for 5 minutes, twice in 0.1X SSC and 0.1% SDS at RT for 10 minutes and four times in 0.1X SSC for 1 minute at RT, and then dried by centrifugation at 1,500 rpm for 2 minutes. Arrays were scanned with an Axon 4000a scanner with fixed PMT (PMT = 550 for Cy3 and 650 for Cy5). Data were acquired and analyzed by Genepix Pro 5.0 (Axon Instrument).

### Statistical analysis

For each developmental stage, dye swaps with two technical replicates and two biological replicates were performed to compensate dye effect and to assess technical and biological reproducibility, leading to eight hybridized slides. Each biological replicate was analyzed separately using R functions (The R project) and Bioconductor package [56]. After logarithm transformation of ratio of the median of the intensities (without background subtraction) in the two channels, an intensity-dependent normalization was applied to each slide. A Loess curve (locally weighted least squares regression) was fitted to (1/2)log2(Cy5×Cy3) versus log2(Cy5/Cy3) plot (MA plot), where 40% of the data was used to calculate the Loess fit at each point [57]. This curve was used to adjust log2(ratio) for each spot. Empty and flagged spots were excluded from the analysis. A paired Student *t* test was used to assess differentially expressed spots. After exclusion of the values presenting too much or not enough variation, the common variance was used for all genes to improve the robustness of the test. The raw *p* values were then corrected using the Bonferroni method with a type I error of 0.05. All log2 ratios are presented as CSA-panned condition over CD36-panned condition. Our data have been submitted to the publicly available ArrayExpress database [58].

### Immunofluorescence

FCR3-CSA and -CD36 *P. falciparum*-infected erythrocytes were taken from asynchronous cultures and processed for indirect immunofluorescence assay as previously described [59]. Slides of air-dried blood films were incubated with the MAb Pf12.8B7.4 [60] for 30 minutes at RT, washed and incubated with Alexa-labeled F(ab') fragment of goat anti-mouse IgG (Molecular Probes) in the same conditions. The nuclei were counterstained with 10 ng/μl DAPI (Molecular Probes). The slides were mounted in 50% glycerol in PBS containing 0.1% p-phenylenediamine (Sigma) as anti-fading. Mouse Mab89 anti-PfHRPI (or PfKAHRP) [61] and guinea pig anti-ATS domain from PfEMP1 (D Mattei, unpublished data) were used as positive controls. Labeled erythrocytes were visualized under UV light in an E800 Nikon Microscope. Images were acquired under identical exposure conditions and processed with Adobe Photoshop 7.0.

### Western blot

Total parasite SDS extracts were subjected to 7.5% SDS-PAGE and were transferred onto nitrocellulose. Membranes were incubated with MAb Pf12.8B7.4 [60] and processed for chemiluminescence detection according to the manufacturer (SuperSignal West Pico Chemiluminescent Substrate, Pierce). Mab1C11 anti-PfHsp70 was used as control [62]. Pre-stained molecular weight markers were obtained from BioRad.

## Additional data files

The following additional data are included with the online version of this article: a table showing normalized array data for all FCR3 and 3D7 sense and antisense oligos included in the analysis, with data from 12 hours (ring stage), 24 hours (trophozoite stage) and 36 hours (early schizont stage) timepoints. The table shows data from biological and dye repeats, in addition to dye swap replicates (Additional data file 1); a table with a subset of the microarray expression data showing normalized array data for the oligos corresponding to sense and antisense strands of *var *genes from 3D7 and FCR3 (Additional data file 2); histograms showing apparent absolute abundance of the *varA4tres *and *varFCR3s1.2 *transcript in CD36 (grey) and CSA (white) panned parasites. Different columns show the apparent absolute abundance for oligonucleotides at individual positions along the genes. Left panels show probes corresponding to sense transcript, right panels show probes corresponding to antisense transcripts. Separate histograms show data for ring, trophozoite and schizont stages. Standard deviation is shown. The antisense patterns for both genes show a pattern that is inconsistent with a *var *silencing role for antisense, with antisense just as high for all life stages in the active population as in the silenced populations. As in other genes, adjacent probes for antisense are much more variable than in the corresponding sense strand, suggesting antisense transcripts are small and interspersed (Additional data file 3); Agilent 2100 bioanalyzer analysis of total RNA used for microarrays. Virtual gel images and electrophereograms are shown for all timepoints for both treatments and replicates (Additional data file 4).

## Supplementary Material

Additional data File 1 A table showing normalized array data for all FCR3 and 3D7 sense and antisense oligos included in the analysis, with data from 12 hours (ring stage), 24 hours (trophozoite stage) and 36 hours (early schizont stage) timepoints. The table shows data from biological and dye repeats, in addition to dye swap replicates Click here for file

Additional data File 2 A table with a subset of the microarray expression data showing normalized array data for the oligos corresponding to sense and antisense strands of *var *genes from 3D7 and FCR3 Click here for file

Additional data File 3 Histograms showing apparent absolute abundance of the *varA4tres *and *varFCR3s1.2 *transcript in CD36 (grey) and CSA (white) panned parasites. Different columns show the apparent absolute abundance for oligonucleotides at individual positions along the genes. Left panels show probes corresponding to sense transcript, right panels show probes corresponding to antisense transcripts. Separate histograms show data for ring, trophozoite and schizont stages. Standard deviation is shown. The antisense patterns for both genes show a pattern that is inconsistent with a *var *silencing role for antisense, with antisense just as high for all life stages in the active population as in the silenced populations. As in other genes, adjacent probes for antisense are much more variable than in the corresponding sense strand, suggesting antisense transcripts are small and interspersed Click here for file

Additional data File 4 Agilent 2100 bioanalyzer analysis of total RNA used for microarrays. Virtual gel images and electrophereograms are shown for all timepoints for both treatments and replicates Click here for file

## References

[B1] Smith JD, Chitnis CE, Craig AG, Roberts DJ, Hudson-Taylor DE, Peterson DS, Pinches R, Newbold CI, Miller LH (1995). Switches in expression of *Plasmodium falciparum **var* genes correlate with changes in antigenic and cytoadherent phenotypes of infected erythrocytes.. Cell.

[B2] Scherf A, Hernandez-Rivas R, Buffet P, Bottius E, Benatar C, Pouvelle B, Gysin J, Lanzer M (1998). Antigenic variation in malaria: *in situ* switching, relaxed and mutually exclusive transcription of *var* genes during intra-erythrocytic development in *Plasmodium falciparum*.. EMBO J.

[B3] Freitas-Junior LH, Hernandez-Rivas R, Ralph SA, Montiel-Condado D, Ruvalcaba-Salazar OK, Rojas-Meza AP, Mancio-Silva L, Leal-Silvestre RJ, Gontijo AM, Shorte S, Scherf A (2005). Telomeric heterochromatin propagation and histone acetylation control mutually exclusive expression of antigenic variation genes in malaria parasites.. Cell.

[B4] Ralph SA, Scheidig-Benatar C, Scherf A (2005). Antigenic variation in *Plasmodium falciparum *is associated with movement of *var *loci between subnuclear locations.. Proc Natl Acad Sci USA.

[B5] Duraisingh MT, Voss TS, Marty AJ, Duffy MF, Good RT, Thompson JK, Freitas-Junior LH, Scherf A, Crabb BS, Cowman AF (2005). Heterochromatin silencing and locus repositioning linked to regulation of virulence genes in *Plasmodium falciparum*.. Cell.

[B6] Horrocks P, Pinches R, Christodoulou Z, Kyes SA, Newbold CI (2004). Variable *var* transition rates underlie antigenic variation in malaria.. Proc Natl Acad Sci USA.

[B7] Peters J, Fowler E, Gatton M, Chen N, Saul A, Cheng Q (2002). High diversity and rapid changeover of expressed *var* genes during the acute phase of *Plasmodium falciparum *infections in human volunteers.. Proc Natl Acad Sci USA.

[B8] Chen Q, Fernandez V, Sundstrom A, Schlichtherle M, Datta S, Hagblom P, Wahlgren M (1998). Developmental selection of var gene expression in. Nature.

[B9] Taylor HM, Kyes SA, Harris D, Kriek N, Newbold CI (2000). A study of *var* gene transcription *in vitro *using universal *var* gene primers.. Mol Biochem Parasitol.

[B10] Su XZ, Heatwole VM, Wertheimer SP, Guinet F, Herrfeldt JA, Peterson DS, Ravetch JA, Wellems TE (1995). The large diverse gene family *var* encodes proteins involved in cytoadherence and antigenic variation of *Plasmodium falciparum*-infected erythrocytes.. Cell.

[B11] Calderwood MS, Gannoun-Zaki L, Wellems TE, Deitsch KW (2003). *Plasmodium falciparum **var* genes are regulated by two regions with separate promoters, one upstream of the coding region and a second within the intron.. J Biol Chem.

[B12] Patankar S, Munasinghe A, Shoaibi A, Cummings LM, Wirth DF (2001). Serial analysis of gene expression in *Plasmodium falciparum *reveals the global expression profile of erythrocytic stages and the presence of anti-sense transcripts in the malarial parasite.. Mol Biol Cell.

[B13] Gunasekera AM, Patankar S, Schug J, Eisen G, Kissinger J, Roos D, Wirth DF (2004). Widespread distribution of antisense transcripts in the *Plasmodium falciparum *genome.. Mol Biochem Parasitol.

[B14] Militello KT, Patel V, Chessler AD, Fisher JK, Kasper JM, Gunasekera A, Wirth DF (2005). RNA polymerase II synthesizes antisense RNA in *Plasmodium falciparum*.. RNA.

[B15] Wanidworanun C, Nagel RL, Shear HL (1999). Antisense oligonucleotides targeting malarial aldolase inhibit the asexual erythrocytic stages of *Plasmodium falciparum*.. Mol Biochem Parasitol.

[B16] Gardiner DL, Holt DC, Thomas EA, Kemp DJ, Trenholme KR (2000). Inhibition of *Plasmodium falciparum **clag9* gene function by antisense RNA.. Mol Biochem Parasitol.

[B17] Noonpakdee W, Pothikasikorn J, Nimitsantiwong W, Wilairat P (2003). Inhibition of *Plasmodium falciparum *proliferation *in vitro *by antisense oligodeoxynucleotides against malarial topoisomerase II.. Biochem Biophys Res Commun.

[B18] Yelin R, Dahary D, Sorek R, Levanon EY, Goldstein O, Shoshan A, Diber A, Biton S, Tamir Y, Khosravi R (2003). Widespread occurrence of antisense transcription in the human genome.. Nat Biotechnol.

[B19] Osato N, Yamada H, Satoh K, Ooka H, Yamamoto M, Suzuki K, Kawai J, Carninci P, Ohtomo Y, Murakami K (2003). Antisense transcripts with rice full-length cDNAs.. Genome Biol.

[B20] Yamada K, Lim J, Dale JM, Chen H, Shinn P, Palm CJ, Southwick AM, Wu HC, Kim C, Nguyen M (2003). Empirical analysis of transcriptional activity in the *Arabidopsis* genome.. Science.

[B21] Reis EM, Nakaya HI, Louro R, Canavez FC, Flatschart AV, Almeida GT, Egidio CM, Paquola AC, Machado AA, Festa F (2004). Antisense intronic non-coding RNA levels correlate to the degree of tumor differentiation in prostate cancer.. Oncogene.

[B22] Cawley S, Bekiranov S, Ng HH, Kapranov P, Sekinger EA, Kampa D, Piccolboni A, Sementchenko V, Cheng J, Williams AJ (2004). Unbiased mapping of transcription factor binding sites along human chromosomes 21 and 22 points to widespread regulation of noncoding RNAs.. Cell.

[B23] Bozdech Z, Llinas M, Pulliam BL, Wong ED, Zhu J, DeRisi JL (2003). The transcriptome of the intraerythrocytic developmental cycle of *Plasmodium falciparum*.. PLoS Biol.

[B24] Smith JD, Kyes S, Craig AG, Fagan T, Hudson-Taylor D, Miller LH, Baruch DI, Newbold CI (1998). Analysis of adhesive domains from the A4VAR *Plasmodium falciparum *erythrocyte membrane protein-1 identifies a CD36 binding domain.. Mol Biochem Parasitol.

[B25] Kyes S, Pinches R, Newbold C (2000). A simple RNA analysis method shows *var* and *rif* multigene family expression patterns in *Plasmodium falciparum*.. Mol Biochem Parasitol.

[B26] Salanti A, Staalsoe T, Lavstsen T, Jensen AT, Sowa MP, Arnot DE, Hviid L, Theander TG (2003). Selective upregulation of a single distinctly structured *var* gene in chondroitin sulphate A-adhering *Plasmodium falciparum *involved in pregnancy-associated malaria.. Mol Microbiol.

[B27] Salanti A, Dahlback M, Turner L, Nielsen MA, Barfod L, Magistrado P, Jensen AT, Lavstsen T, Ofori MF, Marsh K (2004). Evidence for the involvement of VAR2CSA in pregnancy-associated malaria.. J Exp Med.

[B28] Duffy MF, Byrne TJ, Elliott SR, Wilson DW, Rogerson SJ, Beeson JG, Noviyanti R, Brown GV (2005). Broad analysis reveals a consistent pattern of *var *gene transcription in *Plasmodium falciparum *repeatedly selected for a defined adhesion phenotype.. Mol Microbiol.

[B29] Viebig NK, Gamain B, Scheidig C, Lepolard C, Przyborski J, Lanzer M, Gysin J, Scherf A (2005). A single member of the *Plasmodium falciparum **var* multigene family determines cytoadhesion to the placental receptor chondroitin sulphate A.. EMBO Rep.

[B30] Robinson BA, Welch TL, Smith JD (2003). Widespread functional specialization of *Plasmodium falciparum *erythrocyte membrane protein 1 family members to bind CD36 analysed across a parasite genome.. Mol Microbiol.

[B31] Gamain B, Gratepanche S, Miller LH, Baruch DI (2002). Molecular basis for the dichotomy in *Plasmodium falciparum *adhesion to CD36 and chondroitin sulfate A.. Proc Natl Acad Sci USA.

[B32] Miller LH, Hudson-Taylor D, Gamain B, Saul AJ (2002). Definition of the minimal domain of CIDR1alpha of *Plasmodium falciparum *PfEMP1 for binding CD36.. Mol Biochem Parasitol.

[B33] Baruch DI, Ma XC, Singh HB, Bi X, Pasloske BL, Howard RJ (1997). Identification of a region of PfEMP1 that mediates adherence of *Plasmodium falciparum *infected erythrocytes to CD36: conserved function with variant sequence.. Blood.

[B34] Kyes S, Christodoulou Z, Pinches R, Newbold C (2002). Stage-specific merozoite surface protein 2 antisense transcripts in *Plasmodium falciparum*.. Mol Biochem Parasitol.

[B35] Duffy MF, Brown GV, Basuki W, Krejany EO, Noviyanti R, Cowman AF, Reeder JC (2002). Transcription of multiple *var* genes by individual, trophozoite-stage *Plasmodium falciparum *cells expressing a chondroitin sulphate A binding phenotype.. Mol Microbiol.

[B36] Noviyanti R, Brown GV, Wickham ME, Duffy MF, Cowman AF, Reeder JC (2001). Multiple *var* gene transcripts are expressed in *Plasmodium falciparum *infected erythrocytes selected for adhesion.. Mol Biochem Parasitol.

[B37] Costa FT, Fusai T, Parzy D, Sterkers Y, Torrentino M, Douki JB, Traore B, Petres S, Scherf A, Gysin J (2003). Immunization with recombinant duffy binding-like-gamma3 induces pan-reactive and adhesion-blocking antibodies against placental chondroitin sulfate A-binding *Plasmodium falciparum *parasites.. J Infect Dis.

[B38] Lekana Douki JB, Traore B, Costa FT, Fusai T, Pouvelle B, Sterkers Y, Scherf A, Gysin J (2002). Sequestration of *Plasmodium falciparum*-infected erythrocytes to chondroitin sulfate A, a receptor for maternal malaria: monoclonal antibodies against the native parasite ligand reveal pan-reactive epitopes in placental isolates.. Blood.

[B39] Gamain B, Smith JD, Avril M, Baruch DI, Scherf A, Gysin J, Miller LH (2004). Identification of a 67-amino-acid region of the *Plasmodium falciparum *variant surface antigen that binds chondroitin sulphate A and elicits antibodies reactive with the surface of placental isolates.. Mol Microbiol.

[B40] Kyes SA, Christodoulou Z, Raza A, Horrocks P, Pinches R, Rowe JA, Newbold CI (2003). A well-conserved *Plasmodium falciparum **var* gene shows an unusual stage-specific transcript pattern.. Mol Microbiol.

[B41] Marti M, Good RT, Rug M, Knuepfer E, Cowman AF (2004). Targeting malaria virulence and remodeling proteins to the host erythrocyte.. Science.

[B42] Lustigman S, Anders RF, Brown GV, Coppel RL (1990). The mature-parasite-infected erythrocyte surface antigen (MESA) of *Plasmodium falciparum *associates with the erythrocyte membrane skeletal protein, band 4.1.. Mol Biochem Parasitol.

[B43] Magowan C, Coppel RL, Lau AO, Moronne MM, Tchernia G, Mohandas N (1995). Role of the *Plasmodium falciparum *mature-parasite-infected erythrocyte surface antigen (MESA/PfEMP-2) in malarial infection of erythrocytes.. Blood.

[B44] Cooke BM, Glenister FK, Mohandas N, Coppel RL (2002). Assignment of functional roles to parasite proteins in malaria-infected red blood cells by competitive flow-based adhesion assay.. Br J Haematol.

[B45] Le Roch KG, Zhou Y, Blair PL, Grainger M, Moch JK, Haynes JD, De La Vega P, Holder AA, Batalov S, Carucci DJ, Winzeler EA (2003). Discovery of gene function by expression profiling of the malaria parasite life cycle.. Science.

[B46] Gissot M, Refour P, Briquet S, Boschet C, Coupe S, Mazier D, Vaquero C (2004). Transcriptome of 3D7 and its gametocyte-less derivative F12 *Plasmodium falciparum *clones during erythrocytic development using a gene-specific microarray assigned to gene regulation, cell cycle and transcription factors.. Gene.

[B47] Daily JP, Le Roch KG, Sarr O, Fang X, Zhou Y, Ndir O, Mboup S, Sultan A, Winzeler EA, Wirth DF (2004). *In vivo *transcriptional profiling of *Plasmodium falciparum*.. Malar J.

[B48] Gunasekera AM, Patankar S, Schug J, Eisen G, Wirth DF (2003). Drug-induced alterations in gene expression of the asexual blood forms of *Plasmodium falciparum*.. Mol Microbiol.

[B49] Fang J, Zhou H, Rathore D, Sullivan M, Su XZ, McCutchan TF (2004). Ambient glucose concentration and gene expression in *Plasmodium falciparum*.. Mol Biochem Parasitol.

[B50] Wilson RJ (2004). The transcriptome: malariologists ride the wave.. Bioessays.

[B51] Llinas M, DeRisi JL (2004). Pernicious plans revealed: *Plasmodium falciparum *genome wide expression analysis.. Curr Opin Microbiol.

[B52] Trager W, Jensen J (1976). Human malaria parasites in continuous culture.. Science.

[B53] Ginsburg H, Kutner S, Krugliak M, Cabantchik ZI (1985). Characterization of permeation pathways appearing in the host membrane of *Plasmodium falciparum *infected red blood cells.. Mol Biochem Parasitol.

[B54] Bozdech Z, Zhu J, Joachimiak MP, Cohen FE, Pulliam B, DeRisi JL (2003). Expression profiling of the schizont and trophozoite stages of *Plasmodium falciparum *with a long-oligonucleotide microarray.. Genome Biol.

[B55] (2003). ArrayOligoSelector. http://arrayoligosel.sourceforge.net/.

[B56] Gentleman RC, Carey VJ, Bates DM, Bolstad B, Dettling M, Dudoit S, Ellis B, Gautier L, Ge Y, Gentry J (2004). Bioconductor: open software development for computational biology and bioinformatics.. Genome Biol.

[B57] Yang YH, Dudoit S, Luu P, Lin DM, Peng V, Ngai J, Speed TP (2002). Normalization for cDNA microarray data: a robust composite method addressing single and multiple slide systematic variation.. Nucleic Acids Res.

[B58] EMBL-EBI: ArrayExpress. http://www.ebi.ac.uk/arrayexpress.

[B59] Hinterberg K, Scherf A, Gysin J, Toyoshima T, Aikawa M, Mazie JC, da Silva LP, Mattei D (1994). *Plasmodium falciparum*: the Pf332 antigen is secreted from the parasite by a brefeldin A-dependent pathway and is translocated to the erythrocyte membrane via the Maurer's clefts.. Exp Parasitol.

[B60] Howard RJ, Lyon JA, Uni S, Saul AJ, Aley SB, Klotz F, Panton LJ, Sherwood JA, Marsh K, Aikawa M (1987). Transport of an Mr approximately 300,000 *Plasmodium falciparum *protein (Pf EMP 2) from the intraerythrocytic asexual parasite to the cytoplasmic face of the host cell membrane.. J Cell Biol.

[B61] Taylor DW, Parra M, Chapman GB, Stearns ME, Rener J, Aikawa M, Uni S, Aley SB, Panton LJ, Howard RJ (1987). Localization of *Plasmodium falciparum *histidine-rich protein 1 in the erythrocyte skeleton under knobs.. Mol Biochem Parasitol.

[B62] Blisnick T, Lema F, Mazie JC, Pereira da Silva LP (1988). *Plasmodium falciparum*: analysis of B epitopes of a polypeptide antigen expressed in *Escherichia coli*, using monoclonal antibodies.. Exp Parasitol.

