# Supplementary figures and images for: Transcriptome analysis of antigenic variation in Plasmodium falciparum - var silencing is not dependent on antisense RNA
Source: Genome Biol. 2005 Oct 31;6(11):R93. doi: 10.1186/gb-2005-6-11-r93 (PMC1297649; doi:10.1186/gb-2005-6-11-r93)

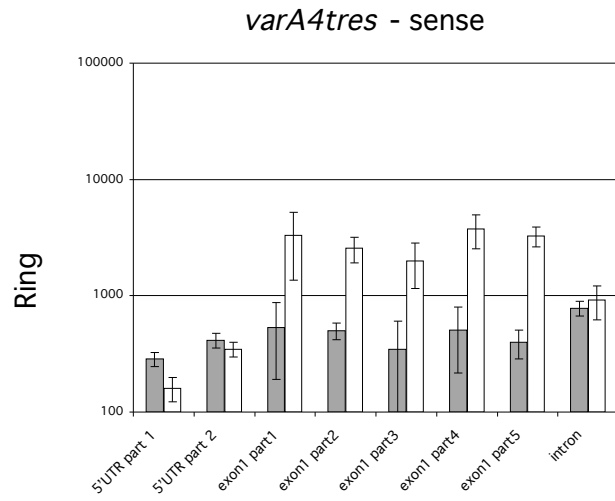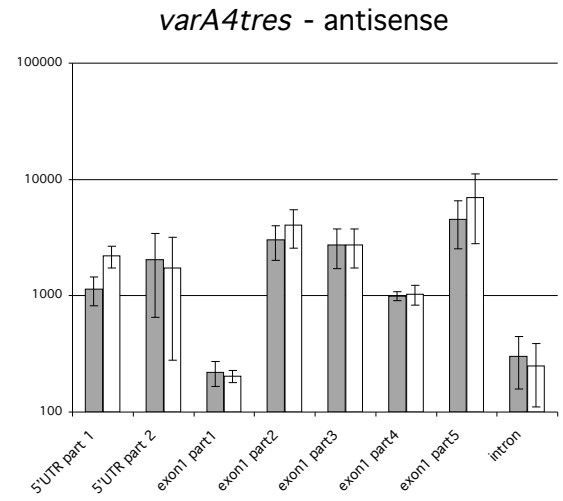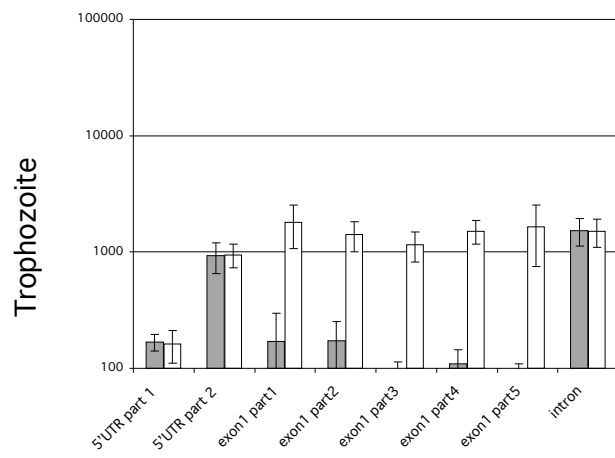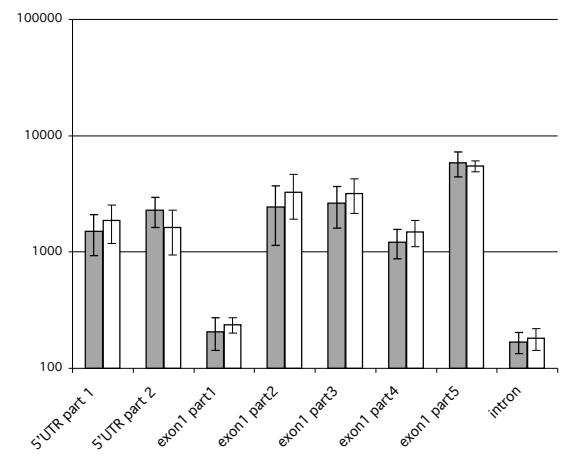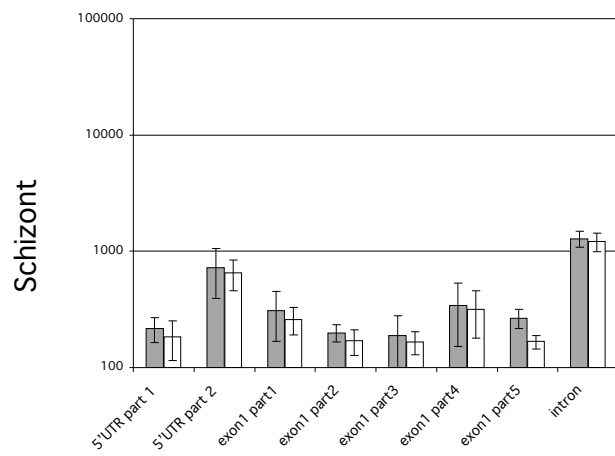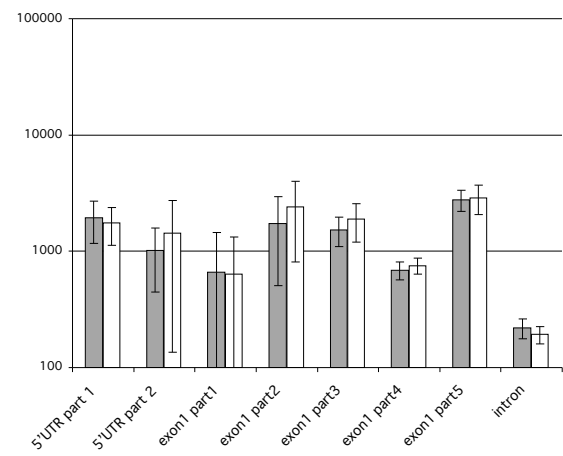

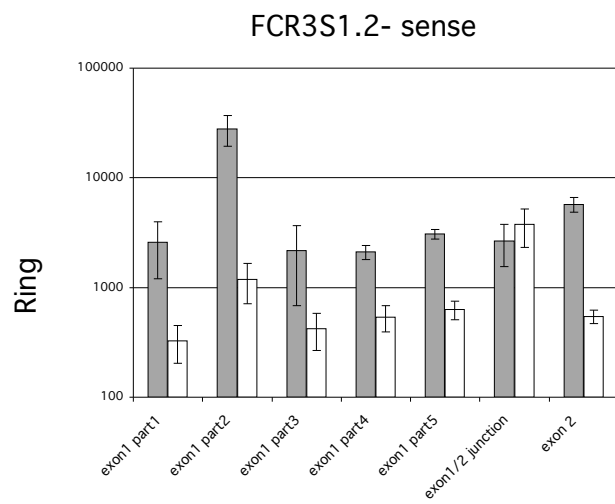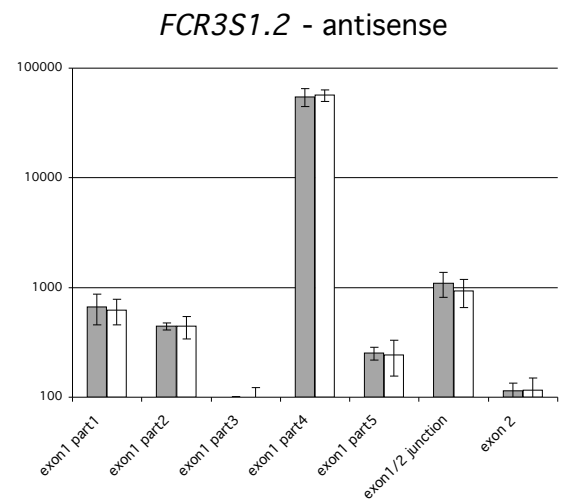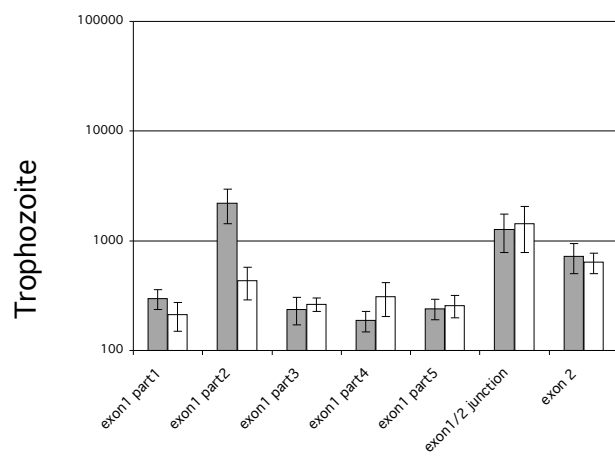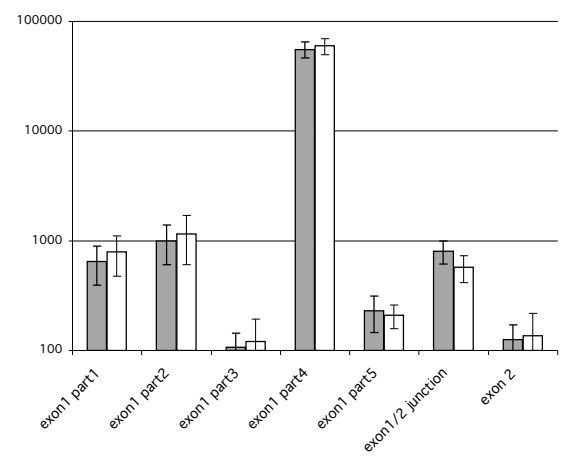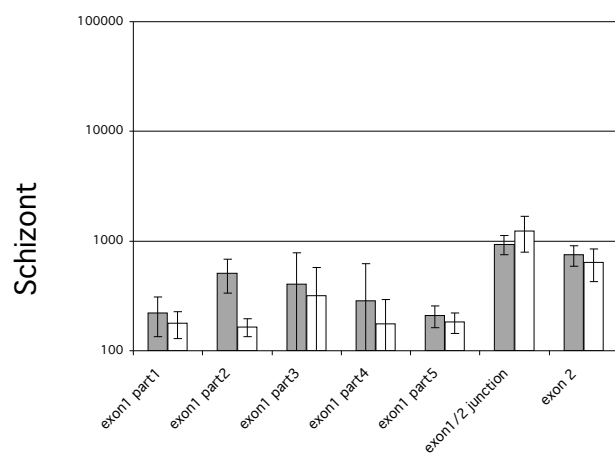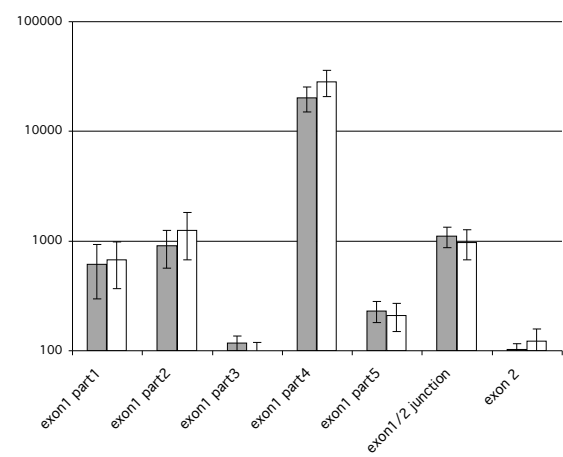

Supplement: Additional data File 3 — Histograms showing apparent absolute abundance of the varA4tres and varFCR3s1.2 transcript in CD36 (grey) and CSA (white) panned parasites. Different columns show the apparent absolute abundance for oligonucleotides at individual positions along the genes. Left panels show probes corresponding to sense transcript, right panels show probes corresponding to antisense transcripts. Separate histograms show data for ring, trophozoite and schizont stages. Standard deviation is shown. The antisense patterns for both genes show a pattern that is inconsistent with a var silencing role for antisense, with antisense just as high for all life stages in the active population as in the silenced populations. As in other genes, adjacent probes for antisense are much more variable than in the corresponding sense strand, suggesting antisense transcripts are small and interspersed [file gb-2005-6-11-r93-S3.pdf]
