# Supplementary material for: Transcriptome analysis of antigenic variation in Plasmodium falciparum - var silencing is not dependent on antisense RNA
Source: Genome Biol. 2005 Oct 31;6(11):R93. doi: 10.1186/gb-2005-6-11-r93 (PMC1297649; doi:10.1186/gb-2005-6-11-r93)
Supplement: Additional data File 4 — Agilent 2100 bioanalyzer analysis of total RNA used for microarrays. Virtual gel images and electrophereograms are shown for all timepoints for both treatments and replicates [file gb-2005-6-11-r93-S4.pdf]

Assay : Eukaryote Total RNA Nano
Data Path : C:/Results Agilent/2004-08-25

Read : 8/25/04 12 :37 :40 PM (A.02.12 SI292)
Modified : 8/25/04 1 :01 :54 PM (A.02.12 SI292)

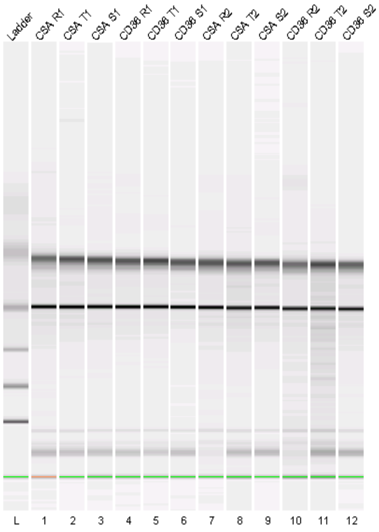

Instrument :	G2938B, Serial# DE23101747, Fireware Version A.01.16
Assay :	C:/Program Files/Agilent 2100 Bioanalyser/Bio
Title :	Eukaryote Total RNA Nano
Version :	1.4
Comments :	Copyright 1999-2002 Caliper Technologies Corp.

Ladder Concentration :	150ng/ul	Baseline :	19 Seconds
Min Peak Height :	0.5 (above baseline)	Filter Width :	1 Seconds
Slope Threshold :	0.8 /Second	Baseline Plateau :	0.5 Seconds
Min Peak Width :	0.5 Seconds	Polynomial Order :	6

Start Time :	20 Seconds
End Time :	69 Seconds

Notes :

CSA Ring bio1

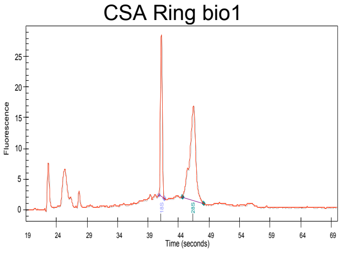

CD36 Troph bio1

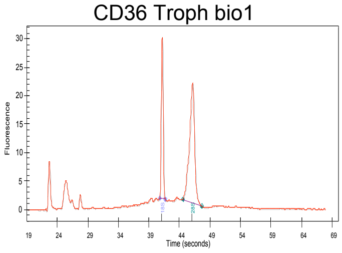

CSA Schiz bio2

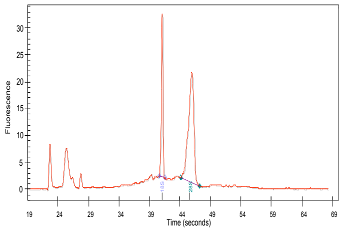

CSA Troph bio1

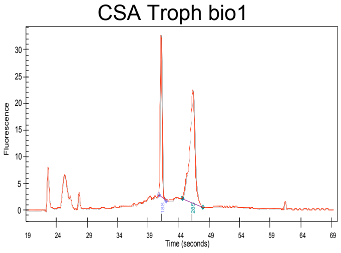

CD36 Schiz bio1

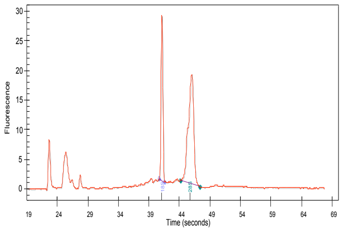

CD36 Ring bio2

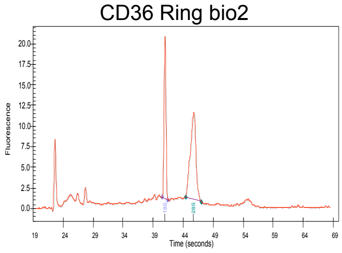

CSA Schiz bio1

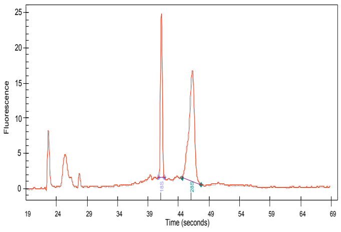

CSA Ring bio2

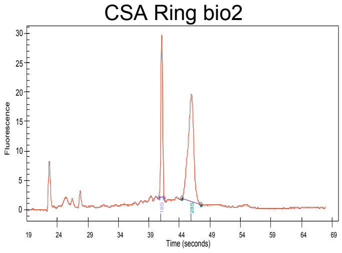

CD36 Troph bio2

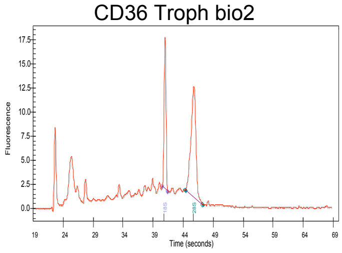

CD36 Ring bio1

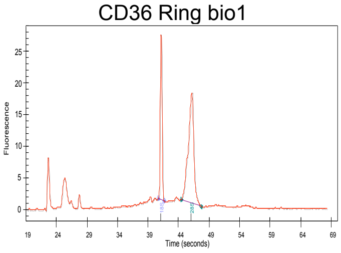

CSA Troph bio2

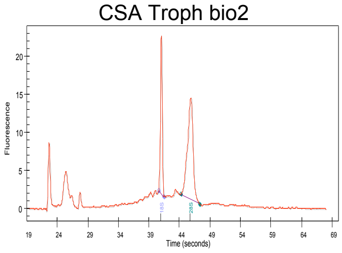

CD36 Schiz bio2

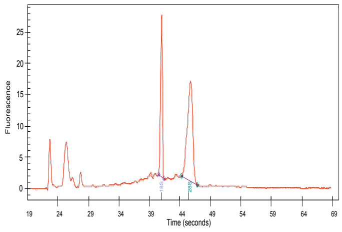

Copyright © 1999-2002, Agilent Technologies, © 1999-2002, Caliper Technologies Corp.

Assay : Eukaryote Total RNA Nano
Data Path : C:/Results Agilent/2004-08-25

Read : 8/25/04 12 :37 :40 PM (A.02.12 SI292)
Modified : 8/25/04 1 :01 :54 PM (A.02.12 SI292)

CSA R1

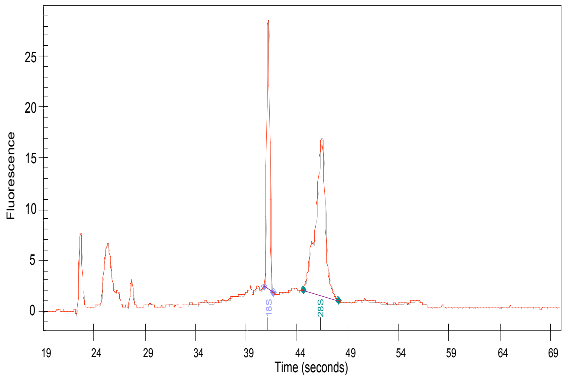

Fragment	Name	Start_Time(secs)		End_Time(secs)	
	Area	%_of_total_Area			
1	18S	40.75	41.65	23.16	14.43
2	28S	44.60	48.05	37.46	23.33

RNA Area 160.53
RNA Concentration(ng/ul) 165.23
rRNA Ratio [28S / 18S] 1.62

CD36 R1

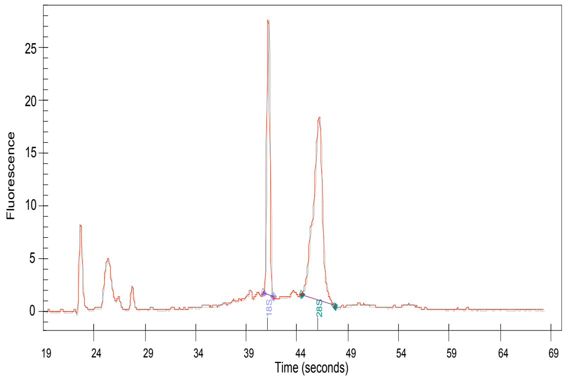

Fragment	Name	Start_Time(secs)		End_Time(secs)	
	Area	%_of_total_Area			
1	18S	40.70	41.65	23.05	17.47
2	28S	44.45	47.70	40.21	30.47

RNA Area 131.97
RNA Concentration(ng/ul) 135.83
rRNA Ratio [28S / 18S] 1.74

CSA T1

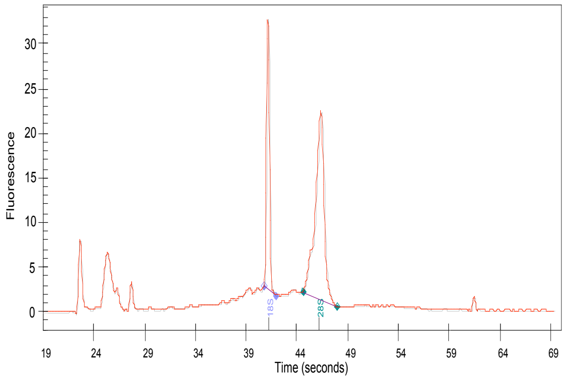

Fragment	Name	Start_Time(secs)		End_Time(secs)	
	Area	%_of_total_Area			
1	18S	40.75	41.90	26.95	15.17
2	28S	44.60	47.95	46.75	26.32

RNA Area 177.65
RNA Concentration(ng/ul) 182.84
rRNA Ratio [28S / 18S] 1.73

CD36 T1

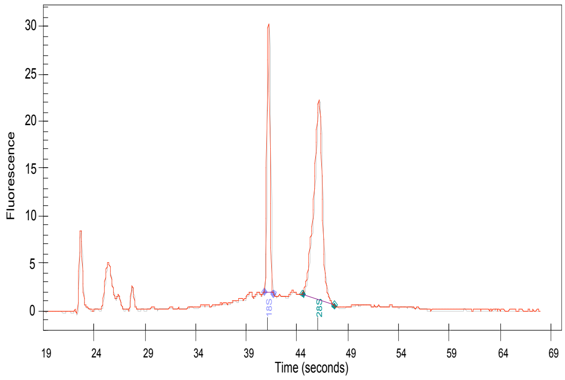

Fragment	Name	Start_Time(secs)		End_Time(secs)	
	Area	%_of_total_Area			
1	18S	40.75	41.65	24.91	16.65
2	28S	44.55	47.65	44.77	29.94

RNA Area 149.56
RNA Concentration(ng/ul) 153.93
rRNA Ratio [28S / 18S] 1.80

CSA S1

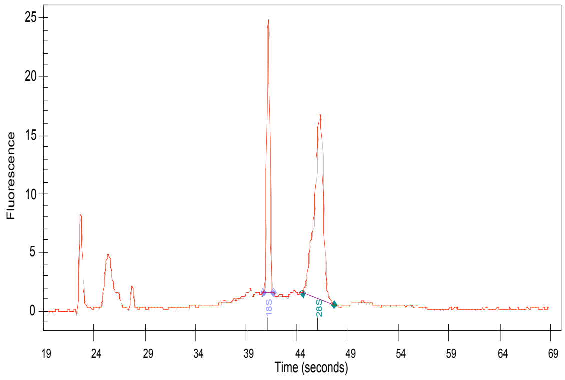

Fragment	Name	Start_Time(secs)		End_Time(secs)	
	Area	%_of_total_Area			
1	18S	40.70	41.65	20.50	15.88
2	28S	44.55	47.65	35.93	27.83

RNA Area 129.12
RNA Concentration(ng/ul) 132.90
rRNA Ratio [28S / 18S] 1.75

CD36 S1

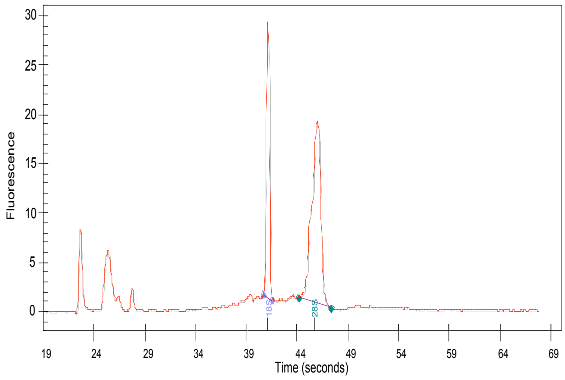

Fragment	Name	Start_Time(secs)		End_Time(secs)	
	Area	%_of_total_Area			
1	18S	40.70	41.55	24.58	18.95
2	28S	44.15	47.35	42.62	32.85

RNA Area 129.74
RNA Concentration(ng/ul) 133.53
rRNA Ratio [28S / 18S] 1.73

Copyright © 1999-2002, Agilent Technologies, © 1999-2002, Caliper Technologies Corp.

Assay : Eukaryote Total RNA Nano | Read : 8/25/04 12 :37 :40 PM (A.02.12 SI292)
Data Path : C:/Results Agilent/2004-08-25 | Modified : 8/25/04 1 :01 :54 PM (A.02.12 SI292)

CSA R2

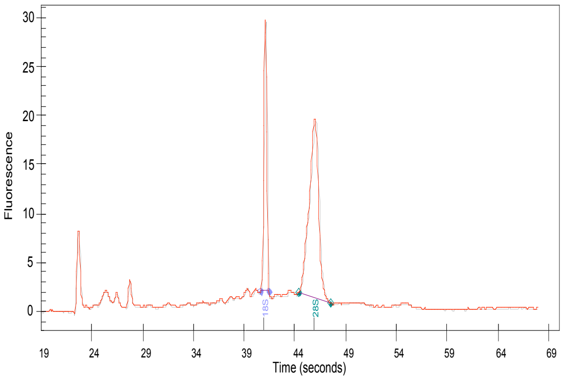

Fragment	Name	Start_Time(secs)		End_Time(secs)	
	Area	%_of_total_Area			
1	18S	40.65	41.45	24.52	16.76
2	28S	44.40	47.50	44.02	30.10

RNA Area 146.26
RNA Concentration(ng/ul) 150.54
rRNA Ratio [28S / 18S] 1.80

CD36 R2

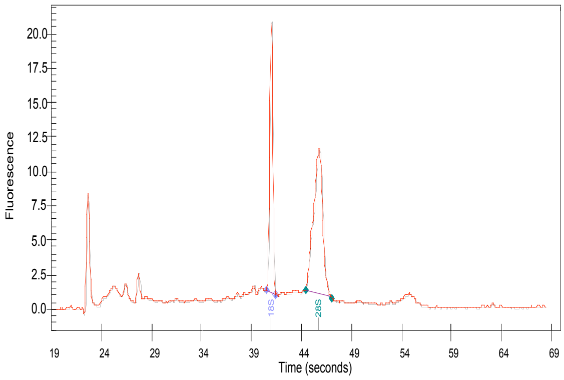

Fragment	Name	Start_Time(secs)		End_Time(secs)	
	Area	%_of_total_Area			
1	18S	40.50	41.40	17.67	15.77
2	28S	44.40	47.00	24.92	22.24

RNA Area 112.02
RNA Concentration(ng/ul) 115.29
rRNA Ratio [28S / 18S] 1.41

CSA T2

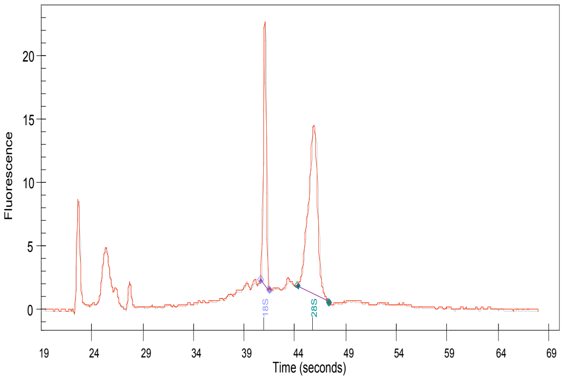

Fragment	Name	Start_Time(secs)		End_Time(secs)	
	Area	%_of_total_Area			
1	18S	40.60	41.45	1833.53	13.73
2	28S	44.25	47.30	31.02	22.98

RNA Area 135.00
RNA Concentration(ng/ul) 138.95
rRNA Ratio [28S / 18S] 1.67

CD36 T2

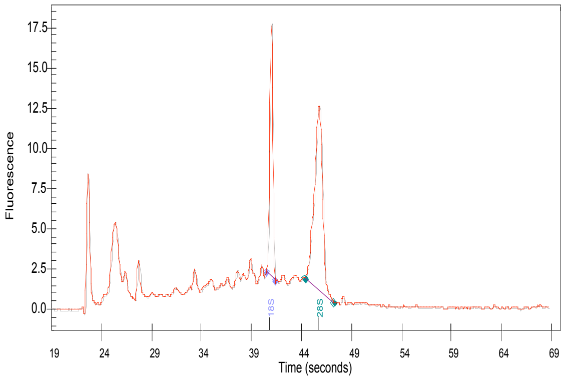

Fragment	Name	Start_Time(secs)		End_Time(secs)	
	Area	%_of_total_Area			
1	18S	40.50	41.35	14.45	9.67
2	28S	44.35	47.25	24.02	16.07

RNA Area 149.46
RNA Concentration(ng/ul) 153.83
rRNA Ratio [28S / 18S] 1.66

CSA S2

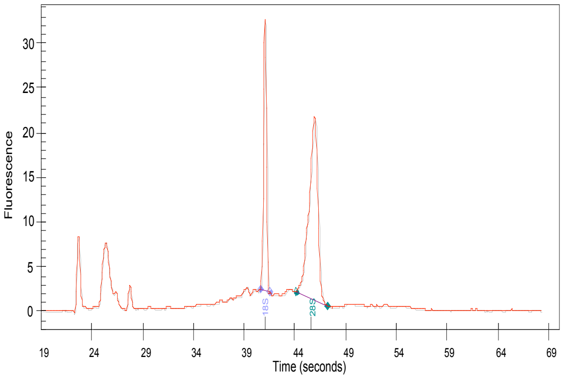

Fragment	Name	Start_Time(secs)		End_Time(secs)	
	Area	%_of_total_Area			
1	18S	40.60	41.55	26.46	14.96
2	28S	44.15	47.20	45.29	25.60

RNA Area 176.93
RNA Concentration(ng/ul) 182.10
rRNA Ratio [28S / 18S] 1.71

CD36 S2

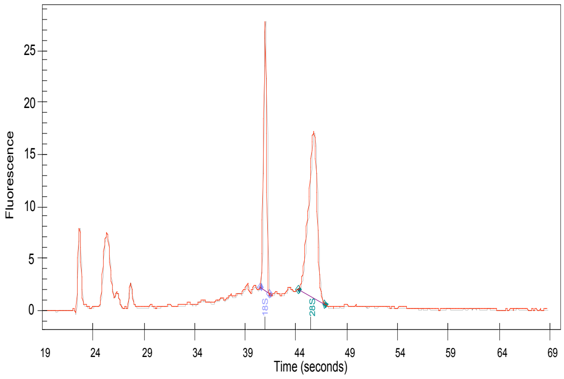

Fragment	Name	Start_Time(secs)		End_Time(secs)	
	Area	%_of_total_Area			
1	18S	40.50	41.40	23.03	15.14
2	28S	44.25	46.85	34.86	22.92

RNA Area 152.10
RNA Concentration(ng/ul) 156.55
rRNA Ratio [28S / 18S] 1.51

Copyright © 1999-2002, Agilent Technologies, © 1999-2002, Caliper Technologies Corp.
